# Managing a Pigmented Corneal Ulcer in a 58-Year-Old Patient

**DOI:** 10.7759/cureus.46850

**Published:** 2023-10-11

**Authors:** Alejandro Acosta, Estefania Ramirez Marquez, Angel Aguayo, Alejandro Perez, Lília Rivera, Armando L Oliver

**Affiliations:** 1 Ophthalmology, University of Puerto Rico, Medical Sciences Campus, San Juan, USA; 2 Ophthalmology and Cornea, University of Puerto Rico, Medical Sciences Campus, San Juan, USA

**Keywords:** corneal disease, ophthalmology, dematiaceous fungi, pigmented corneal ulcer, fungal keratitis

## Abstract

We report on a case study involving a 58-year-old male with a pigmented corneal ulcer. The patient presented with a two-month history of an unresolved corneal ulcer in the oculus sinister (OS), accompanied by increasing ocular discomfort. His best corrected visual acuity (BCVA) was 20/20 oculus dexter and hand motion OS. Examination of OS revealed mild conjunctival injection, diffuse corneal edema, and the presence of a central pigmented lesion. Microbiological analysis via culture identified the causative agent as Ochroconis fungi, belonging to the dematiaceous species. Subsequently, the patient's condition was managed through a comprehensive regimen that included multiple topical antifungal agents, a topical antibiotic, and povidone-iodine drops. After two months of treatment, the patient exhibited improvement in his condition. His BCVA improved to counting fingers at a distance of two feet OS.

## Introduction

Fungal keratitis presents a clinical challenge due to its sight-threatening potential [1,2]. The most common fungi involved are Fusarium and Aspergillus, followed by the dematiaceous species [1-3]. The dematiaceous fungi are associated with vegetative material and with the production of brown pigment [1]. Furthermore, keratitis caused by this species may be uniquely characterized by the presence of brown- and/or olive-colored plaques that are tinted by the previously mentioned pigment [1,4]. These plaques are thought to interfere with the optimal absorption of topical antifungal eye drops, therefore rendering the management of this condition quite challenging [4]. 

The different management strategies described in the literature include the use of topical or intracameral antifungals, keratectomy, and keratoplasty [1-4]. We hereby present the case of a Hispanic male with a pigmented ulcer of dematiaceous species origin, specifically Ochroconis, that was confirmed by cultures. His disease was managed with multiple topical antifungals, a topical antibiotic, and povidone-iodine drops.

## Case presentation

A 58-year-old male was referred to the clinic following a two-month history of a non-resolving corneal ulcer and progressive discomfort in the oculus sinister (OS). The patient reported that the discomfort began after sunblock and sweat got in his eye while he was working in his yard. He denied any previous trauma to the affected eye. His medical history included hypertension and diabetes mellitus, both of which were well-controlled with medication. His ocular history was remarkable for recurrent herpes simplex keratitis OS, from the age of seven years, treated with trifluridine 1% ophthalmic solution and prednisolone ophthalmic solution, as needed. His review of systems and his social and family histories were otherwise unremarkable. 

**Figure 1:**
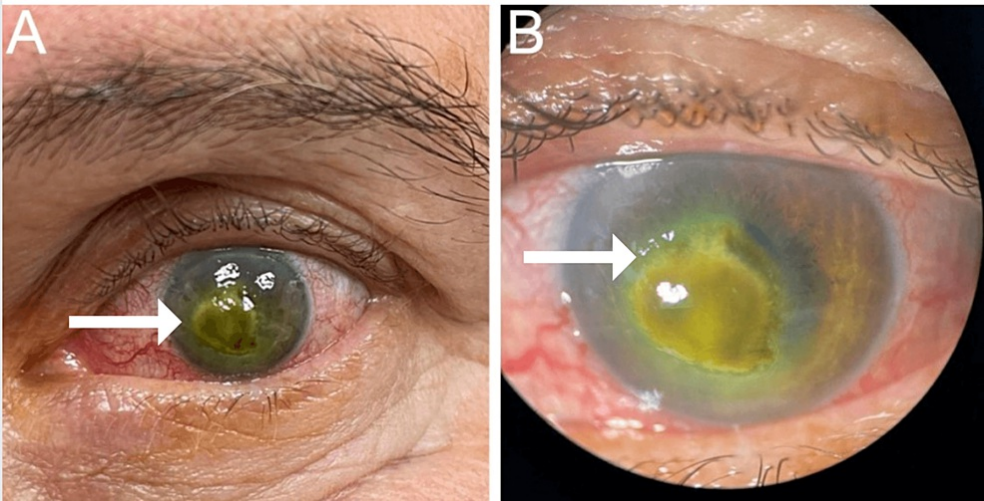
External examination and slit-lamp view of the left eye. External examination (A) and slit-lamp view (B) of the left eye with fluorescein-highlighted diffuse conjunctival injection and central pigmented keratitis.

Upon a comprehensive ophthalmic evaluation, his best-corrected visual acuity (BCVA) was 20/20 in the oculus dexter (OD) and hand motion (HM) OS. The intraocular pressure (IOP), measured with tono-pen, was 11 mmHg OD and 12 mmHg OS. Using reverse testing for relative afferent pupillary defect, the pupils were round and reactive to light, and there was no afferent pupillary defect. An Ishihara color plate test of both eyes revealed no color vision deficiency OD; however, it could not be assessed OS due to the patient's poor vision. Extraocular movements were within normal limits. A slit-lamp examination was unremarkable OD; the same examination, but OS, was remarkable for mild conjunctival injection, diffuse corneal edema, minimal hypopyon (2.1 mm vertically by 2.5 mm horizontally), and a central pigmented lesion measuring 3.7 mm vertically by 4.3 mm horizontally (Figure 1). The lesion did not allow a clear view of the anterior chamber. The patient's right fundus was unremarkable; however, the left fundus could not be assessed. 

The diagnosis of a pigmented corneal ulcer (PCU) OS with a suspected fungal etiology was made. The patient was started on amphotericin 0.5% ophthalmic solution, every two hours, ofloxacin 0.3% ophthalmic solution, voriconazole 1% ophthalmic solution, and povidone-iodine, one drop, four times a day, OS. A corneal culture OS was obtained by collecting corneal scrapings using a sterile blade and subsequently transferring it to an appropriate culture medium. The culture revealed Ochroconis fungi growth. The patient's treatment regimen was maintained to provide broad coverage against both fungal infections and gram-negative bacteria.

At his one-week follow-up, the patient’s BCVA remained 20/20 OD and HM OS. A slit-lamp examination revealed minimal conjunctival injection, diffuse edema, a central pigmented lesion, and a minimal hypopyon (1.7 mm vertically by 1.5 mm horizontally). The following week, the patient complained of increased pain OS. His slit-lamp examination was unchanged compared to that of the previous week; however, the IOP was 30 mmHg OS. The patient was subsequently started on dorzolamide 2%-timolol 0.5% ophthalmic solution, twice daily OS, and brimonidine 0.15% ophthalmic solution, three times daily OS. 

Two months following his initial diagnosis, the patient continued to complain of blurred vision OS. The patient's BCVA remained 20/20 OD and improved to counting fingers at two feet OS. His IOP was 11 mmHg OD and 17 mmHg OS. A slit-lamp examination revealed minimal conjunctival injection, diffuse edema, no pigment, a 1 mm defect, and a hyphema covering the lower third of the anterior chamber. Subsequently, the patient continued to follow-up with a provider outside of the institution.

## Discussion

Pigmented corneal ulcers are a rare occurrence in clinical practice [3]. Due to the rarity of this disease, there is a lack of data on its epidemiology, the risk factors associated with it, and optimal management strategies [1,2]. This condition shares certain features with other corneal disorders, including fungal keratitis and bacterial ulcers [2]. The unique characteristic of a PCU involves the presence of pigmented cells within the layer of the cornea that lead to discoloration and progressive visual impairment [1-4]. The patient discussed in this report presented with a BCVA of HM and a central pigmented lesion, both OS. 

The etiology of PCU is not fully understood; nevertheless, the available literature suggests that chronic inflammation, a history of trauma, and exposure to tropical environments may pose unique risk factors [1,3,4]. This patient had a history of living in a tropical area and a history of a possible microtrauma OS while working with vegetative material in his yard. Furthermore, his medical history was remarkable for diabetes mellitus and hypertension. Diabetes mellitus has been described to induce the dysregulation of innate immunity associated with an increased inflammatory response and could possibly contribute as an additional risk factor for the development of a PCU [2,5]. 

Fusarium and Aspergillus are the most frequently implicated species in cases of PCU [1-3]. In this case, the cultures revealed Ochroconis fungi. Ochroconis is part of the species of dematiaceous that may be isolated from vegetative material. Dematiaceous fungal keratitis poses a challenge in clinical management because it may present with pigmented plaques, such as those described in our patient, which can hinder effective topical drug penetration [4]. There is no established treatment for this disease; nevertheless, there are case reports that present the use of topical natamycin and intracameral amphotericin B, among others [1-4]. This patient was treated with amphotericin 0.5% ophthalmic solution, every two hours, ofloxacin 0.3% ophthalmic solution, voriconazole 1% ophthalmic solution, and povidone-iodine, one drop, four times a day, OS. His vision improved to counting fingers at two feet OS after two months of treatment. Continuing to research this condition is crucial for advancing treatment algorithms, as PCU poses a significant risk to a patient's vision and, in severe cases, may lead to the necessary evisceration or enucleation of the affected eye.

## Conclusions

Ochroconis is a dematiaceous fungus that may cause ocular diseases such as PCUs. This case suggests that environmental exposure, a history of microtrauma, and chronic inflammation secondary to underlying diseases, such as diabetes mellitus, may be risk factors for the development of PCUs. Further studies are needed for the development of improved treatments for PCUs. 
